# Supplementary material for: Efficacy of Different Dosing Regimens of IgE Targeted Biologic Omalizumab for Chronic Spontaneous Urticaria in Adult and Pediatric Populations: A Meta-Analysis
Source: Healthcare (Basel). 2022 Dec 19;10(12):2579. doi: 10.3390/healthcare10122579 (PMC9778713; doi:10.3390/healthcare10122579)
Supplement: Supplementary file 1 [file healthcare-10-02579-s001.zip › healthcare-2007013-supplementary.pdf]

Supplementary Materials

Supplementary S1: Keyword Strings.

Search: **(chronic urticaria) AND (Omalizumab)**	("chronic urticaria"[MeSH Terms] OR ("chronic"[All Fields] AND "urticaria"[All Fields]) OR "chronic urticaria"[All Fields]) AND ("omalizumab"[MeSH Terms] OR "omalizumab"[All Fields] OR "omalizumab s"[All Fields])
**chronic urticaria:**	“chronic urticaria"[MeSH Terms] OR ("chronic"[All Fields] AND "urticaria"[All Fields]) OR "chronicurticaria"[All Fields]
**Omalizumab:**	"omalizumab"[MeSH Terms] OR "omalizumab"[All Fields] OR "omalizumab's"[All Fields]

Supplementary S2: Data Sheet.

ID and Name	Author, Year	Dosage and TimePeriod	Inclusion	Mean Age	Females (IG vs CG)	Race (IG vs CG)	Weekly Itch Score (IG vs CG)	Weekly Wheal Score(IG vs CG)	Responders (IG vs CG)	UAS7 (IG vs CG)
NCT00481676,XCUISITE	Maurer, 2011	Omalizumab was dosed at 75 to 375 mg, subcutaneously every 2 or 4 weeks ending at 24 weeks	Males/Females (18-70 years old) with CU with IgE autoantibodies against thyroperoxidase who had persistent symptoms (wheals and pruritus) despite standard antihistamine therapy	40.5	19/27 (70.4%) vs. 19/22 (86.4%)	All White	**150 mg:** -5.9 (4.43) N=8 vs. -3.57 (4.95) N=22 **300 mg:** -11.19 (6.46) N=8 vs. -3.57 (4.95) N=22	**150 mg:** -7.19 (5.39) N=8 vs. -3.36 (4.34) N=22 **300 mg:** -8.53 (7.01) N=8 vs. -3.36 (4.34) N=22	**150 mg:** 1/7 vs. 1/22 **300 mg:** 5/7vs. 1/22	**75-375 mg:** -17.8 (10.52) N=27 vs. -5.8 (11.52) N=22
NCT01292473,ASTERIA I	Maurer, 2013	Omalizumab 75 mg or 150 mg or 300 mg, subcutaneously every 4 weeks ending at 12 weeks	Patients between the ages of 12 and 75 years with moderate-to-severeCIU who remainedsymptomatic despite H1-antihistamine therapy (licensed doses)	42.5 ± 13.7	189/243 (77.8%) vs. 55/79 (70%)	White: 202/243 (83.1%) vs. 70/79 (89%) Non-White: 31/243 (12.8%) vs. 6/79 (8%) NA: 10/243 (4.1%) vs. 3/79 (4%)	**75 mg:** -6.46 (6.14) N=70 vs. -3.63 (5.22) N=80 **150 mg:** -6.66 (6.28) N=87 vs. -3.63 (5.22) N=80 **300 mg:** -9.4 (5.73) N=81 vs. -3.63 (5.22) N=80	**75 mg:** -7.36 (7.52) N=70 vs. -4.37 (6.6) N=80 **150 mg:** -9.8 (7.3) N=82 vs. -5.2 (6.6) N=79 **300 mg:** -11.35 (7.25) N=81 vs. - 4.37 (6.6) N=80	**75 mg:** 9/77 vs. 7/80 **150 mg:** 12/80 vs. 7/80 **300 mg:** 29/81 vs. 7/80	**75 mg:** -13.08 (12.67) N=82 vs. - 10.36 (11.61) N=79 **150 mg:** -17.89 (13.23) N=82 vs. - 10.36 (11.61) N=79 **300 mg:** -21.74 (12.78) N=79 vs. - 10.36 (11.61) N=79
NCT01287117,ASTERIA II	Saini, 2015	Omalizumab 75 mg or 150 mg or 300 mg subcutaneously every 4 weeks during a 24 week treatment period	Patients aged 12–75 years with CIU/CSU who remained symptomatic despite treatment with approved doses of H1 antihistamines	41.15	179/238 (75.2%) vs. 52/80 (65%)	White: 199/238 (83.6%) vs. 64/80 (80%) Black: 23/238 (9.7%) vs. 10/80 (12.5%) Other: 16/238 (6.7%) vs. 6/80 (7.5%)	**75 mg:** -5.9 (6.5) N=82 vs. -5.1 (5.6) N=79 **150 mg:** -8.1 (6.4) N=82 vs. -5.1 (5.6) N=79 **300 mg:** -9.8 (6) N=79 vs. -5.1 (5.6) N=79	**75 mg:** -7.2 (7) N=82 vs. -5.2 (6.6) N=79 **150 mg:** -9.8 (7.3) N=82 vs. -5.2 (6.6) N=79 **300 mg:** -12 (7.6) N=79 vs. -5.2 (6.6) N=79	**75 mg:** 13/82 vs. 4/79 **150 mg:** 18/82 vs. 4/79 **300 mg:** 35/79 vs. 4/79	**75 mg:** -13.82 (13.26) N=77 vs - 8.01 (11.47) N=80 **150 mg:** -14.44 (12.95) N=80 vs. -8.01 (11.47) N=80 **300 mg:** -20.75 (12.17) N=81 vs. -8.01 (11.47) N=80

NCT01264939, GLACIAL	Kaplan, 2013	Omalizumab 300 mg subcutaneously every 4 weeks during a 24 week treatment period	Patients aged 12 to 75 years old; CIU/CSU for 6 months or longer; itch and hives for more than 6 consecutive weeks before enrollment despite therapy with H1-antihistamines plus H2-antihistamines, LTRAs, or both; UAS7 ≥ 16	43.1 ± 14.1	186/252 (73.8%) vs. 55/83 (66.3%)	White: 223/252 (88.5%) vs. 75/83 (90.4%)	**300 mg:** -10.5 (11.05) N=252 vs. -4.5 (7.7) N=83	**300 mg:** -8.6 (9.82) N=252 vs. -4 (7.42) N=83	**300 mg:** 85/252 vs. 4/83	**300 mg:** -19.01 (13.15) N=252 vs. -8.5 (11.71) N=83
NCT01599637, MOA	Metz, 2019	300 mg, Omalizumab administered subcutaneously every 4 weeks through 85 days	CSU patients (18-75 years) who remained symptomatic despite H-1 antihistamine treatment at approved doses, characterized by the re-occurrence of itch and hives for > 6 weeks before baseline; UAS7 ≥ 16; a CSU diagnosis > 6 months; be on an approved dose of an H1-antihistamine for CSU	39.3	18/20 (90%) vs. 8/10 (80%)	All White	**300 mg:** -11.5 (4.54) N=17 vs. -3.1 (6.04) N=8	**300 mg:** -11.6 (5.68) N=17 vs. -3.3 (8.15) N=8	**300 mg:** 9/25 vs. 0/21	**300 mg:** -23.1 (12.94) N=17 vs. -8.1 (14.45) N=8
NCT01723072, X-ACT	Staubach, 2015	300 mg, Omalizumab once a month via subcutaneous injection for a 28-week treatment period	Patients with CSU aged 18–75 years, with wheals; > 4 occurrences of angioedema in the last 6 months; symptomatic despite high-dose sg H1-antihistamine treatment (2-4 times	42.9 ± 12.3	30/44 (68.2%) vs. 33/47 (70.2%)	White: 42/44 (95.5%) 46/47 (97.9%) Asian: 1/44 (2.3%) vs. 1/47 (2.1%) Other: 1/44 (2.3%) vs. 0/47 (0%)	**300 mg:** -8.3 (7.58) N=44 vs. -2.2 (8.99) N=47	**300 mg:** -8.1 (9.32) N=44 vs. -2.1 (9.43) N=47	**300 mg:** 85/252 vs. 4/83	**300 mg:** -16.8 (14.8) N=44 vs. -6.5 (13.4) N=47

			the approved dose)							
NCT00130234, MYSTIQUE	Saini, 2011	A single subcutaneous doseof 75, 300, or 600 mg of Omalizumabfor a total of 24 weeks	Patients aged 12 to 75 yearswith a history of CIU (>3 months) without a clearly defined cause; moderate-to-severe CIU (pruritus and hives for >3 days in a 7- day period for >6 consecutive weeks) despite treatment with an approved dose of an H1-antihistamine	40.8	44/69 (63.8%) vs. 17/21 (81%)	White: 57/69 (82.6%) vs. 18/21 (85.7%) Black/African American: 6/69 (8.7%) vs. 2/21 (9.5%) Asian: 4/69 (5.8%) vs. 1/21 (4.8%) American Indian or Alaska Native: 2/69 (2.9%) vs. 0/21 (0%)	**75 mg:** -4.5 (5.84) N=23 vs. -3.45 (5.22) N=21 **300 mg:** -9.22 (5.98) N=25 vs. -3.45 (5.22) N=21 **600 mg:** -6.46 (5.63) N=21 vs. -3.45 (5.22) N=21	**75 mg:** -5.28 (6.91) N=23 vs. -3.46 (5.17) N=21 **300 mg:** -10.71 (6.75) N=25 vs. -3.46 (5.17) N=21 **600 mg:** -8.1 (6) N=21 vs. -3.46 (5.17) N=21	NR	**75 mg:** -9.79 (11.75) N=23 vs. -6.91 (9.84) N=21 **300 mg:** -19.93 (12.38) N=25 vs. -6.91 (9.84) N=21 **600 mg:** -14.56 (10.17) N=21 vs. -6.91 (9.84) N=21
NCT01713725	Serrano-Candelas,2017	Omalizumab 300 mg, Subcutaneouslyfor 14 weeks, with 5 total doses	CSU patients being treated with Omalizumab, representing a median disease duration of 6.7 years	44 ± 12.2	8/17 (47.1%) vs. 14/22 (63.6%)	NR	**300 mg:** -1.36 (1.62) N=17 vs. -1 (1.59) N=17	**300 mg:** -1.36 (1.62) N=17 vs. -0.72 (0.79) N=17	NR	**300 mg:** -2.4 (2.5) N=17 vs. -1.2 (2.6)N=17
NCT03328897	Bi, 2021	Omalizumab 150 or 300 mg, injected, every 4 weeks	Children with CU with a duration of over 6 weeks; the onset of symptoms were at least twice or 2 days per week,with the duration ofeach attack within the last 24 hours	8.6	62/108 (57.4%) vs. 55/105 (52.4%)	NR	**150 mg:** -9.66 (0.424) N=167 vs. -5.87 (0.604) N=83 **300 mg:** -10.11 (0.430) N=167 vs. -5.87 (0.604) N=83	NR	**150 mg:** 144/167 vs. 59/83 **300 mg:** 142/167 vs. 59/83	**150 mg:** -20.74 (0.882) N=167 vs. -11.62 (1.258) N=83 **300 mg:** -21.82 (0.895) N=167 vs. -11.62 (1.258) N=83
NCT02329223, POLARIS	Hide,2017	Omalizumab 150 or 300 mg subcutaneously every 4 weeks for12 weeks	Males and females, aged 12 to 75 years, with a CSU diagnosis for 6 months refractory to conventional	43.57	83/144 (57.6%) vs. 48/74 (64.9%)	Japanese: 69/144 (47.9%) vs. 36/74 (48.6%) Korean: 75/144	**150 mg:** -8.8 (0.591) N=70 vs. -6.51 (0.581) N=74 **300 mg:** -10.22 (0.571)	**150 mg:** -9.3 (0.709) N=70 vs. -6.27 (0.696) N=74 **300 mg:** -10.71 (0.684) N=73 vs. -	**150 mg:** 13/70 vs. 3/74 **300 mg:** 26/73 vs. 3/74	**150 mg:** -18.79 (1.288) N=70 vs. 13.9 (1.265) N=74 **300 mg:** -22.44 (1.243) N=73 vs. -13.9 (1.265) N=74

			H1AH at time of randomization			(52.1%) vs. 38/74 (51.4%)	N=73 vs. -6.51 (0.581) N=74	6.27 (0.696) N=74		
